# Diagnostic utility of salivary gland ultrasonography in suspected primary Sjögren’s disease: a comparison of OMERACT-based ordinal and summative scoring

**DOI:** 10.1186/s13075-026-03772-3

**Published:** 2026-02-19

**Authors:** José Antonio Peregrina-Rivas, Ana Belén Maroto-Torres, Úrsula Torres-Parejo, Rosa María Ríos-Pelegrina, Enrique Raya-Álvarez, José Hernández-Quero, Carlos Salvatierra-Sánchez, Juan Salvatierra

**Affiliations:** 1 Internal Medicine Department, Hospital Universitario San Cecilio, Av. Del conocimiento s/n 18007, Granada, Spain; 2 Department of Statistics and Operations Research, Faculty of Sciences, Campus Universitario de Fuente Nueva, University of Granada, Granada, Spain; 3 Provincial Intercenter Clinical Management Unit of Pathology of Granada, Granada Hospitals, Granada, Spain; 4 Rheumatology Department, Hospital Universitario San Cecilio, Granada, Spain; 5 Medicine Department, University of Granada, Granada, 18016 Spain; 6 Applied Mathematics Department, University of Granada, Granada, Spain

**Keywords:** Primary sjögren disease, Salivary glands, Ultrasonography, OMERACT, Ordinal score, Sum score, Labial salivary gland biopsy, Autoantibodies

## Abstract

**Background:**

Salivary gland ultrasonography (SGUS) is a promising, non-invasive tool in the diagnostic workup of suspected primary Sjögren’s disease (SjD). This study aimed to compare the diagnostic accuracy of two SGUS scoring methods - an ordinal score (0–6) and a sum score (0–12) - derived from the OMERACT 0–3 grading system, using labial salivary gland biopsy as the reference standard.

**Methods:**

Sixty consecutive patients with suspected primary SjD underwent SGUS of the four major salivary glands. Each gland was graded using the OMERACT 0–3 system. Two composite scores per patient were calculated: (1) ordinal score (maximum 6), considering the two most affected glands and (2) sum score (maximum 12), obtained by adding the scores of all four glands. Diagnostic accuracy was assessed using ROC curve analysis, with histopathology as reference.

**Results:**

Labial biopsy was positive in 23 patients (38.3%). The ordinal score yielded an AUC of 0.687 (95% CI: 0.546–0.828), with 91.9% specificity (95% CI: 83.1–100%) at a threshold ≥ 4. The sum score showed an AUC of 0.683 (95% CI: 0.543–0.824), with 89.2% specificity (95% CI: 79.2–99.2%) at ≥ 5. SGUS scores ≥ 2 (ordinal) and ≥ 3 (sum) were significantly associated with anti-Ro52 antibody positivity (*p* = 0.017 and *p* = 0.007, respectively). At these high-specificity thresholds, labial biopsy could have potentially been deferred in 16.6% (ordinal ≥ 4) and 21.7% (sum score ≥ 5) of biopsy-positive cases, respectively.

**Conclusions:**

Although overall diagnostic performance was modest, SGUS scoring based on OMERACT definitions demonstrated high specificity at selected thresholds. These findings support its potential role as a biopsy-sparing tool in selected patients with high pre-test probability and compatible serological profiles. Prospective studies are needed to validate these results and optimize threshold selection.

**Trial registration:**

not applicable.

**Supplementary Information:**

The online version contains supplementary material available at 10.1186/s13075-026-03772-3.

## Background

Primary Sjögren’s disease (SjD) is a chronic autoimmune disease characterized by lymphocytic infiltration of the exocrine glands, leading to xerostomia and xerophthalmia. However, its clinical spectrum is broad and heterogeneous, including fatigue, arthralgia and systemic involvement, which often delays diagnosis, particularly in the absence of classic sicca symptoms. To improve diagnostic accuracy, several classification criteria have been proposed over time. The 2002 American-European Consensus Group (AECG) criteria combined subjective symptoms with objective glandular test, histopathology and serology, and remained widely used for over a decade [1]. In 2016, the ACR/EULAR collaboration developed revised criteria based on a weighted algorithm that emphasizes objective findings such as anti-Ro/SSA positivity and labial salivary gland biopsy with focus score ≥ 1, thereby improving specificity, especially in early or seronegative disease [2]. Despite its central role in current classification, labial biopsy (first described by Chisholm and Mason) has several limitations: it is invasive, can cause local complications such as bleeding and infections and may yield false-negative results due to the focal distribution of glandular inflammation [3, 4].

Over the last decade, salivary gland ultrasonography (SGUS) has gained increasing recognition as a valuable imaging tool in the diagnostic workup of primary SjD. Early studies, such as those by Salaffi et al. and Milic et al., demonstrated that SGUS could detect structural changes consistent with sialadenitis, with diagnostic performance comparable to that of sialography and salivary gland scintigraphy [5, 6]. Building on this, Luciano et al. showed that SGUS could effectively distinguish primary SjD from undifferentiated connective tissue diseases, reporting a high specificity (98%) but only moderate sensitivity (66%) [7]. Notably, their analysis was based solely on the most severely affected gland, an approach that may fail to capture the multifocal and patchy distribution of glandular involvement that is typical of primary SjD. Around the same time, Delli et al. published a meta-analysis that underscored both the promising diagnostic utility and the methodological heterogeneity of SGUS in the literature. Variability in the number of glands examined, scoring systems applied, and thresholds used to define abnormality limited comparability across studies and hindered widespread adoption of SGUS in classification criteria [8]. Shortly thereafter, Baldini et al. found that a score ≥ 2, assessed using a 0–3 semiquantitative scale derived from the De Vita classification, in the most severely affected gland yielded a specificity of 98% for primary SjD diagnosis, but again at the cost of limited sensitivity (66%) and in the absence of standardized echostructural definitions [9]. A key step in bridging diagnostic imaging with classification frameworks came from Le Goff et al., who evaluated the added value of SGUS in a large cohort of patients with suspected primary SjD. Their analysis showed that including SGUS, defined as a score ≥ 2 in any major salivary gland using a semiquantitative 0–3 scale adapted form De Vita’s classification, into the 2016 ACR/EULAR criteria increased sensitivity from 87.4% to 91.1% without compromising specificity, when compared against the physician’s diagnosis as the reference standard. This finding supported the potential of SGUS as a reliable, non-invasive modality for detecting structural glandular changes. However, the SGUS assessment in that study was still based on the most affected gland and relied on a non-standardized scoring method, underscoring the need for more refined and reproducible approaches. This challenge was met by the development of a standardized scoring system by the Outcome Measures in Rheumatology (OMERACT) ultrasound working group, which developed an international consensus-based scoring system centred on the most reproducible sonographic features of primary SjD: parenchymal inhomogeneity, hypoechoic areas and fibrotic bands. The resulting four-grade scale (0–3) was validated in multicentre cohorts and demonstrated high inter-and intraobserver reliability, alongside good diagnostic accuracy [10, 11]. Although the OMERACT scoring system has gained widespread acceptance, many published studies still define SGUS positivity using a binary threshold applied only to the most severely affected gland, rather than incorporating the full extent of glandular involvement. This single-gland approach may underestimate the multifocal nature of salivary gland involvement and reduce diagnostic sensitivity. Furthermore, few studies have compared such methods directly with labial biopsy as the reference standard and even fewer have applied the OMERACT system using patient-level scoring strategies that incorporate all four major glands.

In this context, we aimed to evaluate the diagnostic performance of two composite OMERACT-based SGUS scoring strategies, a summative score and an ordinal score, in a real-life cohort of patients with suspected primary SjD. Both approaches integrate the number and severity of abnormalities across all four major salivary glands. We hypothesized that high SGUS scores using these systems could accurately identify patients with biopsy-positive disease and thereby support the role of SGUS as a potential biopsy-sparing tool in clinical practice.

## Methods

### Study design and participants

**Fig. 1:**
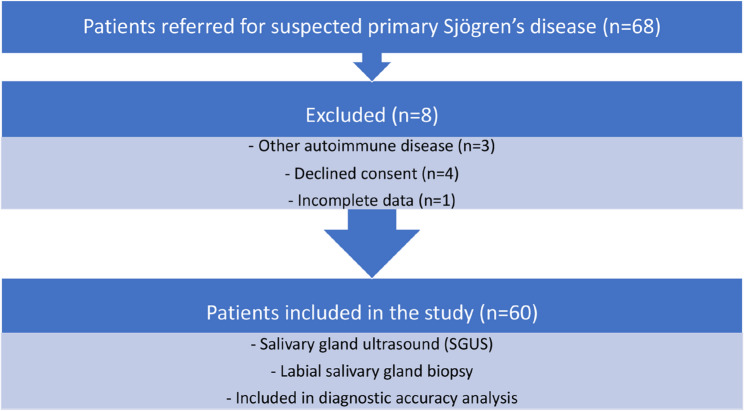
STAD Flow Diagram: Patient Inclusion and Diagnostic Work-Up

This cross-sectional study included 60 consecutive adult patients referred to the Rheumatology Department in 2025 for evaluation of suspected primary Sjögren’s disease (SjD). The study followed the STARD (Standards for Reporting Diagnostic Accuracy Studies) recommendations. Inclusion criteria were: age ≥ 18 years, clinical suspicion of primary SjD (based on ocular/oral symptoms or compatible serology), and indication for minor salivary gland biopsy. Exclusion criteria included previously established primary SjD, diagnosis of other systemic autoimmune diseases, prior salivary gland biopsy or missing key diagnostic data. Given the cross-sectional design and exploratory nature of the study, a convenience sample of consecutive patients was recruited during the study period. A post hoc power analysis is detailed in the Result section. The study flow and diagnostic work-up are detailed in Fig. 1.

All patients underwent standardized clinical assessment, serological testing, unstimulated whole salivary flow rate (sialometry), Schirmer’s test, salivary gland ultrasonography (SGUS) and labial salivary gland biopsy as part of routine diagnostic work-up. SGUS and biopsy were performed within a 4-week interval. The study was approved by the institutional ethics committee (SICEIA-2025-001439), and all participants provided written informed consent before inclusion.

### Clinical and laboratory evaluation

All patients underwent a standardized clinical assessment, including structured anamnesis, Schirmer’s test and unstimulated whole salivary flow rate (sialometry). The Schirmer’s test was performed without anesthesia, using standardized paper strips placed in the lower eyelid for 5 min, with ≤ 5 mm of moisture considered pathological. Unstimulated sialometry was conducted by collecting whole saliva without mechanical or gustatory stimulation over a 5-minute period and expressed in mL/min. Patients were instructed to refrain from eating, drinking, smoking or taking cholinergic medications for at least 90 min before procedure. Serological testing included anti-Ro/SSA and anti-La/SSB antibodies.

### Histopathology

All patients underwent minor labial salivary gland biopsy as part of the diagnostic workup. Tissue specimens were analysed by a single expert pathologist who was blinded to all clinical and imaging data. In addition, the total evaluable glandular area per biopsy was recorded to ensure adequate tissue sampling, given its relevance for focus score reliability. A focus score ≥ 1 per 4 mm² was considered diagnostic, in accordance with the Chisholm and Mason criteria.

### Salivary gland ultrasonography (SGUS)

SGUS was performed using an Esaote MyLab SIGMA ultrasound system (Genova, Italy, 2020) equipped with a 4–15 MHz linear array transducer. Bilateral parotid glands were assessed in both longitudinal and transverse planes, while submandibular glands were examined in the longitudinal plane. Standard technical settings included: gain 57%, depth 4 cm, focus at 2 cm, and dynamic range of 8. These parameters were adjusted as needed in individual patients to optimize gland visualization, depending on body habitus or anatomical variability. Two experienced ultrasonographers, blinded to clinical and histological results, independently evaluated each gland using the OMERACT semiquantitative scoring system (grade 0–3), which considers the presence and extent of key abnormalities, including parenchymal inhomogeneity, hypoechoic areas and fibrotic bands. Discrepancies were resolved by consensus.

### Ultrasound scoring definitions

For each patient, two composite SGUS scores were calculated:

- An ordinal score (range 0–6), designed to reflect both the number and severity of abnormal glands, and defined as follows:
  -  ◦ 0: all glands with OMERACT score 0.
  - ◦ 1: one gland with OMERACT 1, and no glands with 2 or 3.
  - ◦ 2: at least two glands with OMERACT 1, but no glands with 2 or 3.
  - ◦ 3: one gland with OMERACT 2, and no glands with 3.
  - ◦ 4: at least two glands with OMERACT 2, but no glands with 3.
  - ◦ 5: one gland with OMERACT 3.
  - ◦ 6: at least two glands with OMERACT 3.
- A sum score (range 0–12), obtained by adding the OMERACT grades of the four major salivary glands (right and left parotid, right and left submandibular).

Thresholds for diagnostic analysis were defined a priori, based on previous literature and expert consensus: ordinal ≥ 4 and sum ≥ 5 used as high-specificity thresholds for potential biopsy-sparing application.

### Statistical analysis

Statistical analysis was performed using SPSS version 26.0 (IBM Corp., Armonk, NY, USA). Continuous variables were presented as mean ± standard deviation (SD) or median and interquartile range (IQR), as appropriate. Categorical variables were expressed as absolute frequencies and percentages. Between-group comparisons were conducted using the Student’s t-test or Mann-Whitney U test for continuous variables and chi-square or Fisher’s exact test for categorical variables. The Cochran-Armitage trend test was used to assess trends across ordinal SGUS categories.

The level of agreement between the ultrasonographers was assessed using Cohen’s Kappa index, and the Intraclass Correlation Coefficient (ICC) together with Goodman and Kruskal’s Gamma were used to evaluate the association between their assigned scores.

Two SGUS scoring approaches were evaluated: a sum score (range 0–12) and an ordinal score (range 0–6). For each method, receiver operating characteristic (ROC) curves were constructed to assess diagnostic performance for predicting biopsy positivity. The area under the curve (AUC) was calculated and optimal cut-off points were determined using the Youden index as statistical reference. Sensitivity and specificity values were reported for multiple thresholds, along with the corresponding positive and negative likelihood ratios (LR) and their approximate 95% confidence intervals, but clinically prioritizing specificity values. Minor salivary gland biopsy was used as the reference standard. A two-side p-value < 0.05 was considered statistically significant.

As a secondary analysis, ROC curves were also constructed using fulfilment of the 2016 ACR/EULAR classification criteria for primary SjD as the reference standard, and corresponding AUC, sensitivity and specificity values were reported in supplementary material.

## Results

### Sample size justification

A post hoc justification of the sample size was performed based on the observed ROC curve results. The AUC was 0.687 (95% CI: 0.546–0.828), with SE ≈ 0.0727. The AUC was significantly greater than 0.5 (z = 2.57, *p* = 0.010). Post hoc power analysis indicated that the minimum detectable AUC with 80% power (α = 0.05) was approximately 0.682 for a one-tailed test. Accordingly, the available sample size provided adequate power to detect moderate discriminative ability.

### Baseline characteristics

A total of 60 patients with suspected primary SjD were included. Labial salivary gland biopsy was positive (focus score ≥ 1 focus/4 mm²) in 23 patients (38.3%). The mean age of the cohort was 58.2 ± 14.1 years, and 91.7% were women. No significant differences were observed between patients with positive and negative biopsy regarding age, sex, or symptom duration. The proportion of patients with pathologic Schirmer’s test did not differ significantly between groups (47.8% vs. 67.6%, *p* = 0.129). Sialometry values were significantly lower in the biopsy-positive group (0.19 ± 0.22 mL/min vs. 0.33 ± 0.24 mL/min, *p* = 0.036) (Table 1).

**Table 1:** Demographic and clinical characteristics stratified by biopsy result

	Labial salivary gland biopsy		*p*-value
	Positive* (*n* = 23, 38,3%)	Negative (*n* = 37, 61,7%)	
Age (years)	60,1 ± 15,9	57,0 ± 12,5	0,413
Female sex (%)	87,0	94,6	0,575
Symptom duration (months)	46,8 ± 56,0	62,0 ± 48,8	0,303
Schirmer’s test ≤ 5 mm (%)	47,8	67,6	0,129
Sialometry (mL/min)	0,19 ± 0,22	0,33 ± 0,24	**0**,**036**
Anti-Ro52 (n, (%))	14 (60.9%)	5 (13.5%)	**0.000**
Anti-Ro 60 (n, (%))	11 (47.8%)	3 (8.1%)	**0.000**
Anti-La (n, (%))	4 (17.4%)	0 (0%)	**0.009**
Evaluable glandular area per biopsy (mm²)	8.2 ± 1.3	8.4 ± 1.6	0.616

*A positive labial salivary gland biopsy was defined as a focus score ≥ 1 focus/4 mm²

### Ultrasound scores and biopsy results

Cohen’s Kappa was 0.55 (95% CI: 0.42–0.68), indicating a moderate-to-substantial agreement between the ultrasonographers, whereas a Gamma of 0.95 (95% CI: 0.91–0.99) reflects a strong association between their respective scores. The ICC of 0.875 (0.735–0.895) indicates a high degree of agreement or reliability between the two observers.

**Fig. 2:**
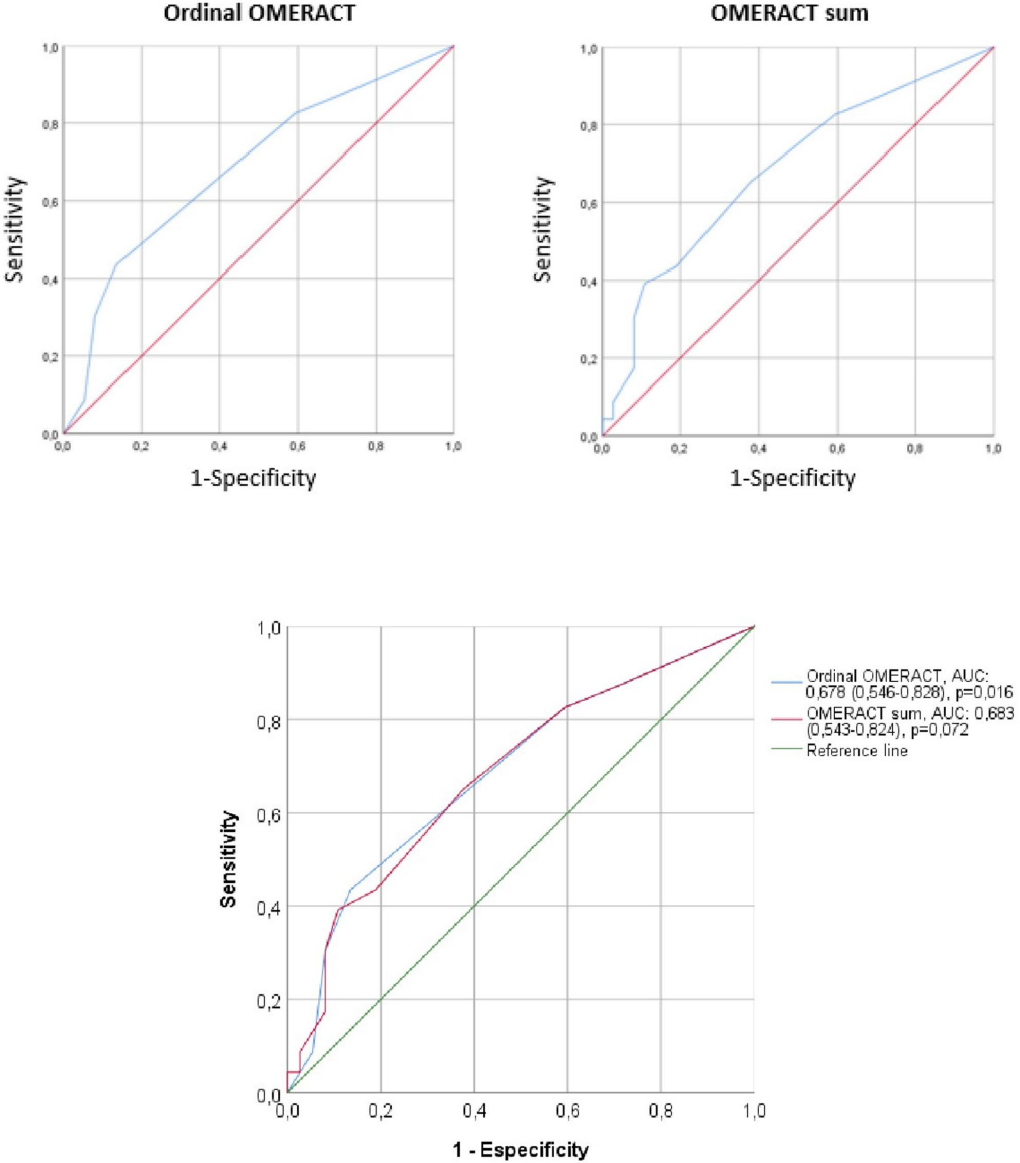
ROC curve analysis of salivary gland ultrasound (SGUS) scores in predicting labial salivary gland biopsy positivity **A** ROC curve for the OMERACT ordinal score (range 0-6); AUC 0.687 (95% CI: 0.546-0.828) **B** ROC curve for the OMERACT sum score (range 0-12); AUC 0.683 (95% CI: 0.543-0.824) **C** Combined ROC curves comparing both scoring strategies. Thresholds ≥4 for the ordinal score and ≥ 5 for the sum score showed the highest specificities (91.9% and 89.2%, respectively)

Ordinal and summative OMERACT scores were calculated for each patient. ROC curve analysis yielded an AUC of 0.687 (95% CI: 0.546–0.828) for the ordinal score and 0.683 (95% CI: 0.543–0.824) for the sum score (Fig. 2). The optimal sensitivity-specificity balance was achieved with an ordinal score ≥ 2 (sensitivity 43.5% (95% CI: 23.3%-63.7%), specificity 86.5% (95% CI: 75.5%-97.5%)) and a sum score ≥ 3 (sensitivity 65.2% (95% CI: 45.7%-85.6%), specificity 62.2% (95% CI: 46.6%-77.8%)), whereas the highest specificities were observed for a score ≥ 5 on the ordinal scale (94.6%, (95% CI: 87.3%-100%)) and ≥ 8 on the sum score (97.3% (95% CI: 92.1%-100%)), and identifying optimal values from a cutoff ≥ 4 on the ordinal scale (91.9% (95% CI: 83.1%-100%) and ≥ 5 on the sum score (89.2% (95% CI: 79.2%-99.2%)). For a cutoff ≥ 2 on the ordinal scale, the LR + is 3.22, indicating that a score ≥ 2 on this scale increases the probability of disease by 3.22 times. On the sum score scale, a cutoff ≥ 5 increases the probability by 3.62 times, and a cutoff ≥ 8 increases it by 4.81 times. (Tables 2 and 3).

**Table 2:** Sensitivity and specificity with ordinal OMERACT, taking lip biopsy as the gold standard

Ordinal OMERACT	Sensitivity (%, (95% CI))	Specificity (%, (95% CI))	Likelihood Ratio + (LR+, 95% CI)	Likelihood Ratio – (LR-, 95% CI)
≥ 0	100 (100–100)	0 (0–0)	1 (1–1)	1 (1–1)
≥ 1	87 (73,3-100)	29,7 (15,0–44,4)	1,24 (1,18 − 1,32)	0,44 (0–1,78)
≥ 2	43,5 (23,3–63,7)	86,5 (75,5–97,5)	3,22 (2,60 − 9,32)	0,65 (0,37 − 1,02)
≥ 3	21,7 (4,8-38-5)	89,2 (79,2–99,2)	2,01 (1,85 − 6,00)	0,88 (0,62 − 1,20)
≥ 4	17,4 (1,9–32,9)	91,9 (83,1-100)	2,15 (1,95 − 19,00)	0,90 (0,67 − 1,18)
≥ 5	8,7 (0–20,2)	94,6 (87,3-100)	1,61 (1,00–1,59)	0,97 (0,80 − 1,14)
≥ 6	4,3 (0–12,6)	100 (100–100)	43 (1,00-126)	0,96 (0,87 − 1,00)

**Table 3:** Sensitivity and specificity with OMERACT sum, taking lip biopsy as the gold standard

OMERACT sum	Sensitivity (%, (95% CI))	Specificity (%, (95% CI))	Likelihood Ratio + (LR+, 95% CI)	Likelihood Ratio – (LR-, 95% CI)
≥ 0	100 (100–100)	0 (0–0)	1 (1–1)	1 (1–1)
≥ 1	87 (73,3-100)	29,7 (15,0–44,4)	1,24 (1,18 − 1,32)	0,44 (0–1,78)
≥ 2	82,6 (67,0–98,2)	40,5 (24,7–56,3)	1,39 (1,30 − 1,53)	0,43 (0,03 − 1,34)
≥ 3	65,2 (45,7–85,6)	62,2 (46,6–77,8)	1,72 (1,60 − 2,06)	0,56 (0,19 − 1,17)
≥ 4	43,5 (23,2–63,8)	81,1 (68,5–93,7)	2,30 (2,03–3,68)	0,70 (0,39 − 1,12)
≥ 5	39,1 (19,2–59,0)	89,2 (79,2–99,2)	3,62 (2,84 − 24,00)	0,68 (0,41 − 1,02)
≥ 6	30,4 (11,6–49,2)	91,9 (83,1-100)	3,75 (2,91–116,00)	0,76 (0,51 − 1,06)
≥ 7	17,4 (1,9–32,9)	94,6 (87,3-100)	3,22 (2,59 − 19,00)	0,87 (0,67 − 1,12)
≥ 8	13 (0–26,7)	97,3 (92,1-100)	4,81 (1,00–33,80)	0,89 (0,73 − 1,08)
≥ 9	8,7 (0–20,2)	97,3 (92,1-100)	3,22 (1,00–25,60)	0,94 (0,80 − 1,08)
≥ 10	4,3 (0–12,6)	97,3 (92,1-100)	1,59 (1,00–1,59)	0,98 (0,87 − 1,08)
≥ 11	4,3 (0–12,6)	100 (100–100)	43 (1,00-126,00)	0,96 (0,87 − 1,00)
≥ 12	0 (0–0)	100 (100–100)	1 (1–1)	1 (1–1)

The ROC curves were also calculated using the ACR/EULAR 2016 classification criteria as an additional reference, yielding sensitivity, specificity, and AUC results for both ordinal and summative OMERACT cutoff points that were similar to those obtained using a positive biopsy as the reference (Supplementary Tables 6–7 and Supplementary Fig. 3).

The correlation of the ordinal and sum score scales with symptom duration is positive, although not significant (Pearson’s *r* = 0.22 and 0.25, respectively).

### Associations with clinical and serological features

An ordinal score ≥ 2 was significantly associated with anti-Ro52 positivity (41.5% vs. 10.5%, *p* = 0.017), with no significant differences in Schirmer’s test or sialometry (Table 4). At higher SGUS thresholds, both ordinal and sum scores were associated with higher frequencies of anti-Ro52, anti-Ro60 and anti-La antibodies (Table 5).

**Table 4:** Antibodies, sialometry and schirmer’s test according to OMERACT ORDINAL cut-off point ≥ 2

Anti-Ro 52	Ordinal OMERACT ≥ 2 (*n* = 41, 68,3%)	Ordinal OMERACT < 2 (*n* = 19, 31,7%)	*p*-value
	17 (41,5%)	2 (10,5%)	0,017
Anti-Ro 60	12 (29,3%)	2 (10,5%)	0,189
Anti-La	4 (9,8%)	0 (0%)	0,297
Sialometry (mL/min)	0,29 ± 0,25	0,24 ± 0,22	0,731
Pathologic Schirmer’s test (%)	20 (64,5%)	16 (55,2%)	0,460

**Table 5:** Antibodies, sialometry and Schirmer's test according to OMERACT sum cut-off point ≥3 and ≥5

Anti-Ro 52	OMERACT sum ≥ 3 (*n* = 29, 48,3%)	OMERACT sum < 3 (*n* = 31, 51,7%)	*p*-value
	14 (48,3%)	5 (16,1%)	0,007
Anti-Ro 60	10 (34,5%)	4 (12,9%)	**0**,**048**
Anti-La	4 (13,8%)	0 (0,0%)	**0**,**049**
Sialometry (mL/min)	0,27 ± 0,23	0,27 ± 0,25	0,949
Pathologic Schirmer’s test (%)	16 (56.5%)	20 (64,5%)	0,319

	OMERACT sum ≥ 5 (*n* = 13, 21,7%)	OMERACT sum < 2 (*n* = 47, 78,3%)	p- value
Anti-Ro 52	9 (69,2%)	10 (21,3%)	**0**,**002**
Anti-Ro 60	7 (53,8%)	7 (14,9%)	**0**,**007**
Anti-La	3 (23,1%)	1 (2,1%)	**0**,**029**
Sialometry (mL/min)	0,30 ± 0,25	0,19 ± 0,18	0,145
Pathologic Schirmer’s test (%)	8 (61,5%)	28 (59,6%)	0,898

### Diagnostic thresholds and biopsy-sparing potential

Based on the distribution of scores, 41 out 60 patients (68.3%) had an ordinal score ≥ 2, including 19 of the 23 patients with biopsy-confirmed primary SjD (82.6%). This threshold could have potentially identified biopsy-positive individuals in whom histological confirmation might have been unnecessary. Similarly, a sum score ≥ 3 and ≥ 5 would have identified 15 (65.2%) and 9 (39.1%) biopsy-positive patients, respectively.

### Diagnostic thresholds and associations with clinical features

We further analyzed whether SGUS thresholds identified as diagnostically informative (based on Youden index) correlated with other clinical features of primary SjD. Patients with an OMERACT ordinal score ≥ 2, identified as the optimal threshold by ROC analysis, had a significantly higher prevalence of anti-Ro52 antibodies compared to those below the threshold (41.5% vs. 10.5%, *p* = 0.017), with no significant differences in sialometry or Schirmer’s test results. Similarly, an OMERACT sum score ≥ 3 was associated with higher frequency of anti-Ro52 positivity and higher frequencies of anti-Ro60 and anti-La (Table 5). The sum score ≥ 5 showed a similar pattern, with progressively higher seropositivity rates across antibodies. No significant differences in salivary flow or Schirmer’s test were observed at any threshold.

## Discussion

The primary aim of this study was to assess the diagnostic utility of two patient-level scoring strategies, derived from the OMERACT definitions for salivary gland ultrasonography (SGUS) in patients with suspected primary SjD: an ordinal scale (0–6) and a summative score (0–12), both integrating the number and severity of affected glands. When compared to labial salivary gland biopsy, both scores showed modest diagnostic performance, with area under the ROC curve (AUC) values of 0.683 (95% CI: 0.543–0.824) for the sum score and 0.687 (95% CI: 0.546–0.828) for the ordinal scale. Nevertheless, they demonstrated high specificity at certain thresholds (≥ 4 for the ordinal score and ≥ 5 for the sum score), highlighting their potential clinical value as biopsy-sparing tools in selected patients [10, 12, 13].

Previous studies have evaluated SGUS as a diagnostic tool for primary SjD using a variety of scoring systems. Mossel et al. reported good agreement with histopathology using the Hocevar score (AUC 0.82) and a specificity of 85% [14]. Barrio-Nogal et al. applied a 0–4 scale and used the most affected gland to define positivity, achieving 90% sensitivity but lower specificity (67%) [15]. Tang et al. summarized in a 2023 meta-analysis that pooled sensitivity and specificity for SGUS were 81% and 87%, respectively, although reference standards varied, and scoring systems were heterogeneous [16]. Unlike these approaches, we evaluated composite scoring methods based on OMERACT criteria that capture global glandular involvement, providing a reproducible and clinically feasible framework.

Various semiquantitative systems have been proposed to standardize SGUS interpretation in primary SjD. The Hocevar score, though comprehensive, has been criticized for its complexity and limited feasibility in routine practice [17]. Attempts to simplify it, for instance, by focusing solely on hypoechogenic areas, have improved applicability but at the expense of omitting other relevant echostructural features [13]. Cornec et al., proposed a simplified 0–4 scale based on De Vita, which showed high specificity but lacked multicentre validation and standardized reliability testing [18]. In contrast, the OMERACT system, developed by international consensus, offers a pragmatic balance between diagnostic value and usability. Its 0–3 scale focuses on reproducible features- parenchymal inhomogeneity, hypoechoic areas and fibrotic bands- and has demonstrated high inter-reader agreement and external validity, fulfilling key OMERACT criteria for clinical application [10, 14, 19].

Our findings reinforce the role of SGUS as a structural imaging biomarker associated with systemic autoimmunity in primary SjD. In our cohort, an OMERACT ordinal score ≥ 2 was significantly associated with anti-Ro52 positivity, while higher thresholds (≥ 3 for ordinal, ≥ 5 for sum score) correlated with anti-Ro52, anti-Ro60 and anti-La antibodies. These results align with previous studies reporting significant associations between abnormal SGUS findings and various autoantibodies, including ANA, anti-SSA and rheumatoid factor positivity [14, 20, 21]. Taken together, this evidence supports the interpretation of SGUS as a non-invasive surrogate marker of B-cell mediated glandular damage in primary SjD.

Although SGUS is emerging as a key structural biomarker in primary SjD, our results, and previous reports, suggest that its findings are not consistently aligned with measures of glandular function. In our cohort, no significant associations were found between SGUS scores and either unstimulated salivary flow or Schirmer’s test. This aligns with prior reports by Schmidt and Mossel, reinforcing the interpretation that SGUS captures chronic structural damage rather than real-time exocrine performance [20, 22]. A recent study by Shi et al. further supported this dissociation, despite only moderate sensitivity, an SGUS grade 3 in the most affected gland and a patient-level sum score ≥ 9 (OMERACT-based) were strongly associated with biopsy positivity and reduced salivary flow, achieving a specificity of 93% [23]. While some studies, such as that by Caraba et al., have noted parallel declines in structure and function, these discrepancies may reflect cohort variability [24]. Altogether, our results highlight the complementary nature of SGUS and functional testing in the diagnostic approach to primary SjD.

This study has several strengths. First, we evaluated two patient-level scoring strategies based on the OMERACT consensus definitions, an ordinal scale (0–6) and a sum score (0–12), that incorporate both the number and severity of abnormalities across all major salivary glands. Second, SGUS readings were performed independently by two experienced operators blinded to clinical and serological data, with discrepancies resolved by consensus. Third, the study included a real-world cohort of consecutive patients referred for suspected primary SjD, increasing its external validity and closely reflecting diagnostic scenarios encountered in daily practice. Finally, the use of labial salivary gland biopsy as the reference standard provides a clinically meaningful comparator. In addition, documenting the evaluable glandular area ensured adequate and homogeneous tissue sampling, thereby reinforcing the reliability and reproducibility of histopathology as the reference standard [25].

Nonetheless, some limitations must be acknowledged. The sample size was moderate and derived from a single-centre cohort, which may limit generalizability. Although interobserver agreement was evaluated using kappa and ICC metrics, the readings were performed by experienced sonographers at a single centre, which may overestimate reproducibility compared to multicentre or less specialized settings. Histological interpretation by a single pathologist ensured internal consistency but may reduce external applicability. Finally, the cross-sectional design captures diagnostic performance at a single time point. In addition, only patients with suspected primary SjD were included, excluding those with previously diagnosed disease or overlapping systemic autoimmune conditions. While this improves internal validity, it may limit extrapolation to broader clinical populations, such as patients with secondary Sjögren’s or more complex immunological profiles. Similarly, although common comorbidities (e.g., hypertension, dyslipidemia) were not exclusion criteria, their influence on SGUS findings was not specially analyzed. Finally, variations in disease duration, symptom burden or pre-test probability may also affect SGUS performance and should be explored in future studies. Longitudinal studies are needed to explore the utility of SGUS scores in disease monitoring, stratification or prognostication.

Our study supports the use of SGUS as a non-invasive, reproducible and clinically informative tool in the diagnostic assessment of patients with suspected primary SjD. By applying two patient-level scoring strategies derived from the OMERACT definitions -an ordinal scale and a sum score- we observed that SGUS abnormalities were frequently aligned with biopsy positivity and serological markers of autoimmunity. Although the overall discriminative performance of both scores was modest (AUC ~ 0.68), their clinical utility lies in the identification of sonographic patterns with high specificity. For example, an OMERACT ordinal score ≥ 4 and a sum score ≥ 5 both achieved specificities close to 92%. In this context, and assuming compatible clinical and serological profiles, these thresholds could potentially be used to avoid biopsy in selected patients with high pre-test probability. This concept is consistent with previous studies suggesting that salivary gland ultrasonography may help reduce the need for labial salivary gland biopsy in selected patients. In particular, Astorri et al. demonstrated that ultrasound abnormalities of the major salivary glands were strong predictors of biopsy positivity in patients with sicca symptoms, supporting the use of SGUS as a stratification tool to prioritize biopsy only in cases with inconclusive or discordant clinical and serological findings [26]. In support of this, the likelihood ratio for a sum score ≥ 5 was 3.62 (95% CI: 2.84-24.00), and for the ordinal score ≥ 4 was 2.15 (95% CI: 1.95-19.00), reinforcing their diagnostic utility as “rule-in” thresholds. Conversely, lower thresholds such as ordinal score ≥ 2 or sum ≥ 3 yielded weaker negative likelihood ratios (LR-), limiting their role in safely ruling out disease. Although these thresholds did not reach absolute specificity, they consistently exceeded 89%, supporting their potential utility as clinically robust biopsy-sparing markers when interpreted alongside compatible clinical and serological features. In our cohort, this approach would have spared biopsy in approximately 16.6% and 21.7% of patients, respectively. Lower thresholds such as ordinal score ≥ 2 or sum score ≥ 3 -though less specific- would have identified a greater proportion of biopsy-positive cases (82.6% and 65.2%, respectively), illustrating the trade-off between diagnostic certainty and test avoidance. These findings underscore the potential value of SGUS as a gatekeeper in the diagnostic algorithm, especially when high-specificity thresholds are applied in patients with concordant clinical and serological features. Thus, the clinical utility of SGUS scoring appears to be limited to “rule-in” applications using high-specificity thresholds, rather than broad screening or exclusion strategies.

Finally, a recent study by Finzel et al. confirmed the good interobserver reliability of the OMERACT scoring system when applied at the patient level, further supporting its broader integration into diagnostic workflows [27]. While promising, these results must be interpreted cautiously given the single-center, cross-sectional design of our study, and should be validated in larger, prospective cohorts.

## Conclusions

Salivary gland ultrasonography scored according to OMERACT definitions -using both patient-level ordinal and sum scores - offers a standardized and reproducible method for evaluating patients with suspected primary SjD. Although the overall diagnostic performance of the scoring systems was modest (AUC ~ 0.68), both strategies demonstrated high specificity at defined thresholds. In particular, an ordinal score ≥ 4 and a sum score ≥ 5 were strongly associated with biopsy positivity and achieved specificities close to 92%. Under these conditions, biopsy could potentially have been deferred in 16.6% and 21.7% of cases, respectively. These findings support the selective use of SGUS as a biopsy-sparing tool in patients with high pre-test probability and compatible serological and clinical features. Further prospective studies are needed to validate these results and define the role of SGUS-based thresholds in future diagnostic algorithms.

## Supplementary Information

Supplementary Material 1.

## Data Availability

The datasets used and analyzed during the current study are available from the corresponding author on reasonable request.

## Abbreviations

ACR: American College of Rheumatology
AECG: American-European Consensus Group
ANA: Antinuclear antibodies
AUC: Area under the curve
CI: Confidence interval
ICC: Intraclass correlation coefficient
IQR: Interquartile range
LR: likelihood ratio
