# Supplementary material for: Diagnostic utility of salivary gland ultrasonography in suspected primary Sjögren’s disease: a comparison of OMERACT-based ordinal and summative scoring
Source: Arthritis Res Ther. 2026 Feb 19;28:77. doi: 10.1186/s13075-026-03772-3 (PMC13019863; doi:10.1186/s13075-026-03772-3)
Supplement: Supplementary file 1 — Supplementary Material 1. [file 13075_2026_3772_MOESM1_ESM.docx]

**Table 6.** Sensitivity and specificity with ordinal OMERACT, using fulfilment of the 2016 ACR/EULAR classification criteria as the reference.

| Ordinal  OMERACT | Sensitivity (%, (95% CI)) | Specificity (%, (95% CI)) | Likelihood Ratio + (LR+, 95% CI) | Likelihood Ratio – (LR-, 95% CI) |
| --- | --- | --- | --- | --- |
| ≥ 0 | 100 (100-100) | 0 (0-0) | 1 (1-1) | 1 (1-1) |
| ≥ 1 | 86,4 (72-100) | 28,9 (14,5-43,4) | 1,22 (1,17-1,27) | 0,47 (0-1,93) |
| ≥ 2 | 81,8 (65,7-97,9) | 39,5 (23,9- 55,0) | 1,35 (1,29-1,46) | 0,46 (0,04-1,44) |
| ≥ 3 | 45,5 (24,6-66,3) | 86,8 (76,1-97,6) | 3,45 (2,77-10,25) | 0,63 (0,35-0,99) |
| ≥ 4 | 31,8 (12,4-51,3) | 92,1 (83,5-100) | 4,03 (3,11-124,00) | 0,74 (0,49-1,05) |
| ≥ 5 | 9,1 (0-21,1) | 94,7 (87,6-100) | 1,72 (1,00-1,70) | 0,96 (0,79-1,14) |
| ≥ 6 | 4,5 (0-13,2) | 97,4 (92,3-100) | 1,73 (1,00-1,71) | 0,98 (0,87-1,08) |

**Table 7.** Sensitivity and specificity with OMERACT sum, using fulfilment of the 2016 ACR/EULAR classification criteria as the reference.

| OMERACT  sum | Sensitivity (%, (95% CI)) | Specificity (%, (95% CI)) | Likelihood Ratio + (LR+, 95% CI) | Likelihood Ratio – (LR-, 95% CI) |
| --- | --- | --- | --- | --- |
| ≥ 0 | 100 (100-100) | 0 (0-0) | 1 (1-1) | 1 (1-1) |
| ≥ 1 | 86,4 (72-100) | 28,9 (14,5-43,4) | 1,22 (1,17-1,27) | 0,47 (0-1,93) |
| ≥ 2 | 81,8 (65,7-97,9) | 39,5 (23,9-55) | 1,35 (1,29-1,46) | 0,46 (0,04-1,44) |
| ≥ 3 | 63,6 (43,5-83,7) | 60,5 (45-76,1) | 1,61 (1,52-1,82) | 0,60 (0,21-1,26) |
| ≥ 4 | 40,1 (20,4-61,5) | 78,9 (66-91,9) | 1,90 (1,81-2,52) | 0,76 (0,42-1,21) |
| ≥ 5 | 40,1 (20,4-61,5) | 89,5 (79,7-99,2) | 3,82 (3,03-25,5) | 0,67 (0,39-1,00) |
| ≥ 6 | 31,8 (12,4-51,3) | 92,1 (83,5-100) | 4,03 (3,11-124,00) | 0,74 (0,49-1,05) |
| ≥ 7 | 18,2 (2,1-34,4) | 92,1 (83,5-100) | 2,30 (2,08-21,00) | 0,89 (0,66-1,17) |
| ≥ 8 | 13,6 (0-28) | 94,7 (87,6-100) | 2,57 (2,26-10,00) | 0,91 (0,72-1,14) |
| ≥ 9 | 9,1 (0-21,1) | 97,4 (92,3-100) | 3,50 (2,74-10,00) | 0,93 (0,79-1,08) |
| ≥ 10 | 4,5 (0-13,2) | 97,4 (92,3-100) | 1,73 (1,00-1,71) | 0,98 (0,87-1,08) |
| ≥ 11 | 4,5 (0-13,2) | 100 (100-100) | 45 (1,00-132,00) | 0,96 (0,87-1,00) |
| ≥ 12 | 0 (0-0) | 100 (100-100) | 1 (1-1) | 1 (1-1) |

**Figure 3.** ROC curve analysis of salivary gland ultrasound (SGUS) scores for predicting fulfilment of the 2016 ACR/EULAR classification criteria. (A) ROC curve for the OMERACT ordinal score (range 0-6); AUC 0,688 (95% CI: 0,544-0,831). (B) ROC curve for the OMERACT sum score (range 0-12); AUC 0,669 (95% CI: 0,524-0,813). (C) Combined ROC curves comparing both scoring strategies. Thresholds ≥4 for the ordinal score and ≥ 5 for the sum score showed the highest specificities (92,1% and 89,5%, respectively).


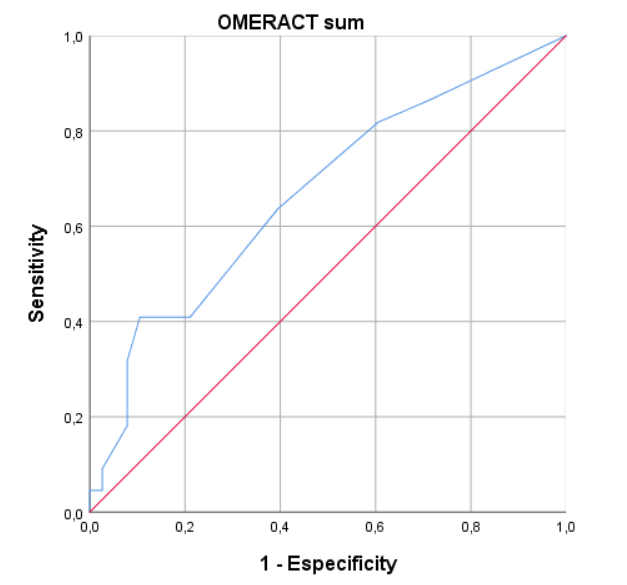

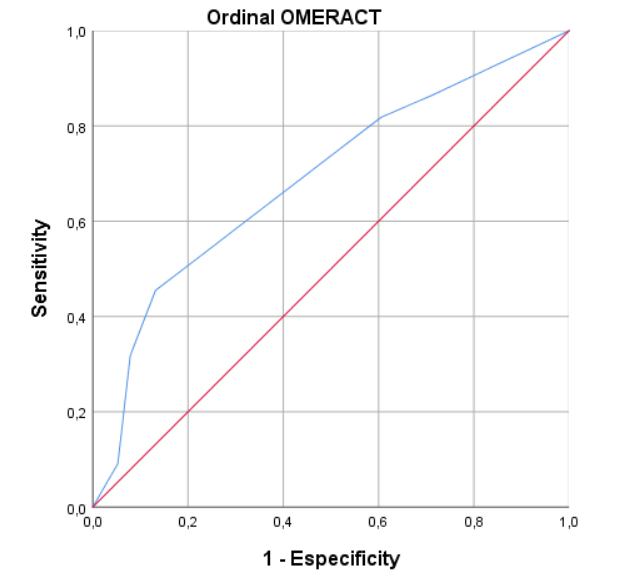

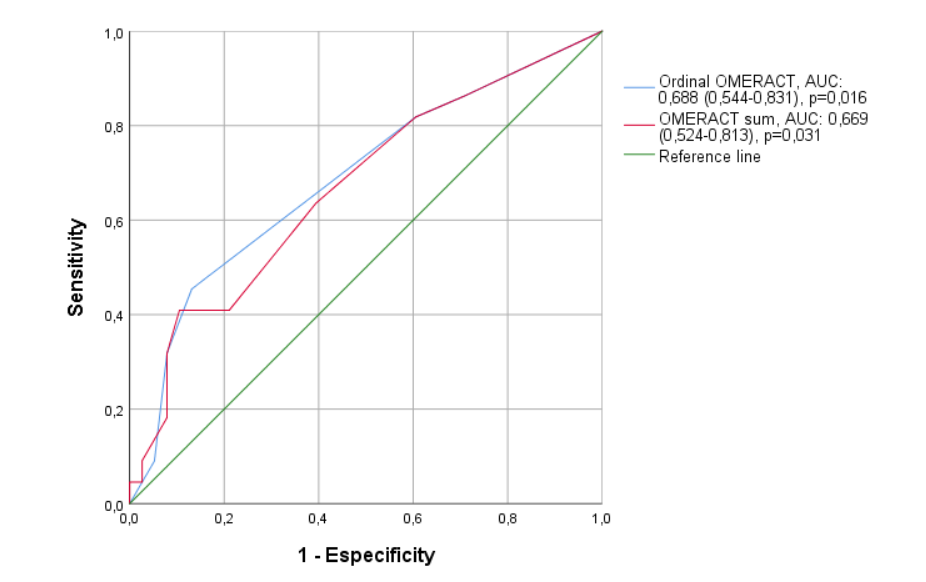


**C**

**B**

**A**
